# Morphogenetic dispositions for variability in acute kidney injury after cardiac surgery: Pilot study

**DOI:** 10.3389/fmed.2022.943254

**Published:** 2022-09-16

**Authors:** Radmila Karan, Natasa Kovačević-Kostić, Bratislav Kirćanski, Jelena Čumić, Duško Terzić, Vladimir Milićević, Vojislav Velinović, Miloš Velinović, Biljana Obrenović-Kirćanski

**Affiliations:** ^1^Faculty of Medicine, University of Belgrade, Belgrade, Serbia; ^2^Department of Anesthesiology and Intensive Care at Clinic for Cardiac Surgery, Clinical Center of Serbia, Belgrade, Serbia; ^3^Pacemaker Center, Clinical Center of Serbia, Belgrade, Serbia; ^4^Department for Transplantation and LVAD at Clinic for Cardiac Surgery, Clinical Center of Serbia, Belgrade, Serbia; ^5^Clinic for Cardiac Surgery, Clinical Center of Serbia, Belgrade, Serbia; ^6^Clinic for Cardiology, Clinical Center of Serbia, Belgrade, Serbia

**Keywords:** coronary artery disease, observable recessive human traits, acute kidney injury, variability, cardiac surgery

## Abstract

**Background:**

The aim of our study was to evaluate the degree of genetic homozygosity in cardiac surgical patients with postoperative acute kidney injury (AKI), compared to the subgroup without postoperative AKI, as well as to evaluate antropomorpho-genetic variability in cardiac surgical patients with regard to the presence and severity degree of AKI.

**Materials and methods:**

The prospective cohort study included an analysis of 138 eligible coronary artery disease (CAD) surgical patients that were screened consecutively. The tested group was divided into three subgroups according to RIFLE criteria: Subgroup NoAKI (*N* = 91), risk (*N* = 31), and injury (*N* = 16). All individuals were evaluated for the presence of 19 observable recessive human traits (ORHT) as a marker of chromosomal homozygosity and variability.

**Results:**

Comparing subgroups NoAKI and risk, four ORHTs were significantly more frequent in the risk subgroup. Comparing subgroups NoAKI and injury, nine ORHTs were significantly more frequent in the injury subgroup; while comparing the injury subgroup and risk, five ORHTs were significantly more frequent in injury than in the risk subgroup. Results also showed a significant increase in the mean value of ORHTs for the injury subgroup compared to NoAKI subgroup (*p* = 0.039). Variability decreased proportionally to the increase in the severity of AKI (V_*NoAKI*_ = 32.81%, V_*Risk*_ = 30.92%, and V_*Injury*_ = 28.62%).

**Conclusion:**

Our findings pointed to the higher degree of recessive homozygosity and decreased variability in AKI patients vs. NoAKI individuals, thus presumably facilitating the development and severity degree expression of AKI in patients after cardiac surgery.

## Introduction

Acute kidney injury (AKI) is a very common and serious complication of cardiac surgery procedures with the use of a cardiopulmonary bypass machine (CPB) affecting 23.2% of patients (1). It is a well-known risk-factor for morbidity and mortality in cardiac surgery patients. The AKI after cardiac surgery can vary from a mild degree of renal impairment to the need for renal replacement therapy. There are different classification and staging systems for the diagnosis of AKI—The RIFLE criteria include changes in serum creatinine levels from baseline or decrease in estimated glomerular filtration rate (eGFR), AKIN criteria—a slight modification of RIFLE criteria, that does not utilize change in GFR, KDIGO that has omitted eGFR changes and uses only changes in SCR levels from baseline and urine output (RIFLE, AKIN, and KDIGO). The value of eGFR reflects the number of function nephrons. The SCr value is changed only after 24–48 h of acute injury, and it can remain normal even if eGFR decreases by more than 50%. This could show that a more timely estimate of renal function is obtained using the creatinine clearance, which approximates the GFR (1–5).

The cause of the onset and degree of postoperative AKI is multifactorial including preoperative renal function, perioperative hemodynamic instability, nephrotoxic agents, but also predisposition to the inflammatory response to the use of CPB. Previous studies have shown that CPB is the second most common cause of AKI after Sepsis. Gaudino et al. stressed out that 174G-C polymorphism IL6 correlated with the onset of AKI after the use of CPB (Gaudino), while Stafford-Smith et al. found two allels IL6-572C and angiotensinogen 842C are connected with post-CPB AKI, also polymorphism for APO-E. It is very important to recognize patients with the increased risk of AKI and the severity degree of AKI, in order to take appropriate preventive measures.

Despite the numerous morphophysiological and genetic studies that were previously conducted (6–9), it is still a challenging task to establish expected phenotype variations in not only healthy human individuals but also in diseased groups as well.

We hypothesized that an increased degree of observable recessive homozygosity and decreased variability might be potential biomarkers for the development and severity degree of AKI. Therefore, the aim of our study was two-fold. First, we aimed to evaluate the degree of genetic homozygosity in cardiac surgical patients with postoperative AKI, compared to the subgroup without postoperative AKI. The second aim was to evaluate anthropomorphic-genetic variability in cardiac surgical patients with regard to the presence and severity degree of AKI.

## Materials and methods

### Study group

**FIGURE 1:**
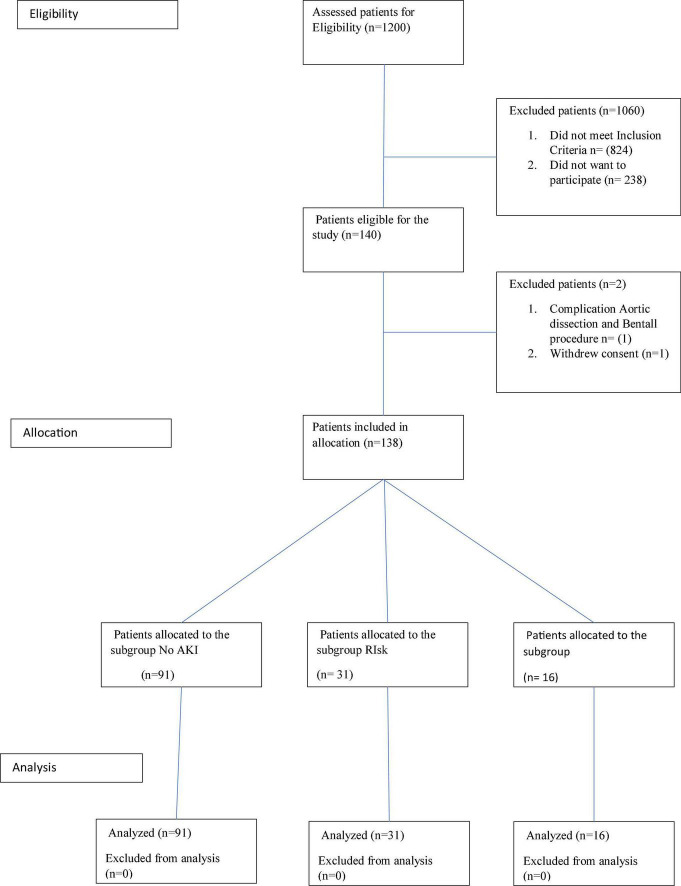
CONSORT diagram: flow diagram graphically describes the design of the study: enrollment, allocation, and data analysis.

The prospective cohort study included eligible CAD patients consecutively screened between 2015 and 2018 and admitted for surgical treatment of CAD at Cardiac Surgery Clinic, Clinical Center of Serbia. Inclusion criteria were: patients 18 years and older, signed Informed consent (ICF), admission for elective Coronary Artery Bypass Surgery (CABG), on pump CABG. Exclusion criteria were: any combined cardiac surgical procedure, urgent or emergency cardiac surgery procedure, and patients with chronic kidney disease on dialysis. There were 140 patients eligible for the study. One patient experienced a complication during the CABG procedure-iatrogenic dissection of the aorta that was immediately managed with Bentall surgery. Due to this complication, the patient was not eligible to continue the study anymore and was excluded (Figure 1).

One patient decided to withdraw consent immediately after surgery and was also excluded. Finally, 138 eligible patients were divided into 3 subgroups regarding the presence and degree of AKI according to the RIFLE criteria (5) and statistically analyzed. These subgroups are subgroup NoAKI (91 patients), subgroup Risk (31 patients), and subgroup injury (16 patients). Considering RIFLE criteria, patients with NoAKI were defined as individuals that had a decrease eGFR postoperatively by less than 25% compared to preoperative eGFR value. Patients with risk were defined as individuals with postoperative decrease in eGFR between 25 and 50%. Patients with injury had postoperative decrease in eGFR by more than 50% (10; Figure 1).

The study followed the principles of good clinical practice and recommendations of the declaration of Helsinki. The study was approved by the relevant institutional Review Board. Prior to inclusion in the study, patients were informed about the study protocol and consent was obtained.

### Tested determinants

All individuals were evaluated for the presence of observable recessive human traits (ORHT) to establish the proportion of clearly expressed ORHTs for every tested individual where one might be considered as a marker of chromosomal homozygosity, pointing out the degree of genetic homozygosity in humans (6–9, 11–15). For the purpose of our study 19 ORHTs were tested in every studied individual. Only ORHTs with extreme appearance was marked as a present trait: attached ear lobe (OMIM number 128900), continuous frontal hair line (OMIM number 194000), blue eyes (gene location 15q12, 15q13, OMIM number 227220; 5p13 OMIM number 227240; 14q32.1, OMIM number 210750; 9q23 OMIM number 612271), straight hair (1q21.3, OMIM number 139450), soft hair and blond hair (gene location 15q12, 15q13, OMIM number 227220; 14q32.1, OMIM number 210750; 12q21.3 OMIM number 611664; 11q13.3, OMIM number 612267), double hair whorl, opposite hair whorl orientation (OMIM number 139400), as well as an inability to roll, fold and curve the tongue (OMIM number 189300), ear without a Darwinian notch, and a guttural “r,” proximal thumb hyperextensibility, index finger longer than the ring finger (OMIM number 136100), left-handedness (gene location 2p12-q22, OMIM number 139900), right thumb over left thumb (hand clasping) (OMIM number 139800), top joint of the thumb > 45°, 3 tendons in the wrist (OMIM) (16).

### Statistical analysis

The results were presented as absolute numbers and percentages, while continuous variables were presented as mean value (MV) ± standard deviation (SD) or median with interquartile range. For comparison between subgroups, we performed Mann–Whitney *U* Test. To test significant predictors of the number of ORHTs between tested subgroups of patients we used univariate logistic regression analysis (ULRA) and multivariate logistic regression analysis (MLRA), in the form of odds ratio (OR) with 95% CI. Variation coefficient (*V*), which is calculated as the ratio of the SD and the mean value expressed as a percentage, was used to compare variability between evaluated groups of individuals. Statistical significance was set at *p* < 0.05.

## Results

Table 1 shows demographic and risk factors frequencies in our patients. There was no statistical difference among risk factors, gender, and age between patients in group NoAKI and patients that developed AKI after surgery (Risk + Injury). No nephrotoxic drugs were used during the intrahospital stay. In Table 2 we presented frequencies of OHRTs among patients with the presence and severity degree of AKI. In the subgroup of NoAKI, the most frequent ORHT was “Inability to longitudinally toung roll” just above half (52.7%), whereas the least frequent was “Double Hair Whorl” (2.2%). Regarding subgroup risk, the most frequent ORHT was “Right Thumb over the Left Thumb”-just above two thirds (67.7%), while the least frequent were “Attached ear-lobe” and “Left-handedness” (3.2%). Patients from subgroup injury had most frequently present “Right Thumb over the Left Thumb”—just above two-thirds (68.8%), while “Attached ear-lobe,” “Left-handedness,” and “Double Hair Whorl” were absent in the studied sample. Patients from subgroup AKI (Injury + Risk) had the most frequently present “Right Thumb over the Left Thumb” and “Straight Hair,” 68.1 and 55.3%, respectively.

**TABLE 1:** Demographic and risk factor frequencies.

Parameters	NoAKI *N* = 91	AKI (Risk + Injury) *N* = 47	*P*-value
Male n (%)	66 (72.5)	30 (63.8)	0.332
Female n (%)	25 (27.5)	17 (36.2)	
Age (mean ± SD)	65.77 ± 7.63	66.02 ± 8.56	0.402
Hypertension n (%)	90 (98.9)	45 (95.7)	0.267
Diabetes mellitus n (%)	45 (49.5)	28 (59.6)	0.284
Hyperlipoproteinemia n (%)	73 (82)	37 (78.7)	0.827
Smoking n (%)	41 (29.7)	24 (17.4)	0.590

**TABLE 2:** Frequencies of observable recessive human traits among patients with presence and severity degree of acute kidney injury.

Observable recessive human traits	NoAKI *N* = 91	Risk *N* = 31	Injury *N* = 16	Risk + Injury *N* = 47
Blond hair	14 (15.4)	12 (38.7)	4 (25)	16 (34)
Straight hair	42 (46.2)	17 (54.8)	9 (56.3)	26 (55.3)
Double hair whorl	2 (2.2)	2 (6.5)	0 (0)	2 (4.3)
Opposite hair whorl orientation	15 (16.5)	7 (22.6)	5 (31.3)	12 (25.5)
Soft hair	23 (25.3)	6 (19.4)	6 (37.5)	12 (25.5)
Continuous frontal hair line	9 (9.9)	2 (6.5)	3 (18.8)	5 (10.6)
Attached ear lobe	10 (11)	1 (3.2)	0 (0)	1 (2.1)
Ear without Darwinian notch	13 (14.3)	4 (12.9)	1 (2.7)	5 (10.6)
Blue eyes	38 (41.8)	14 (45.2)	9 (56.3)	23 (48.9)
Speaking deficiency-gutorral “r”	5 (5.5)	2 (6.5)	1 (6.3)	3 (6.4)
Inability to longitudinally tongue roll	48 (52.7)	8 (25.8)	13 (81.3)	21 (44.7)
Inability to transversally tongue roll	30 (33.3)	9 (29)	4 (25)	13 (27.7)
Right thumb over left thumb	43 (47.3)	21 (67.7)	11 (68.8)	32 (68.1)
Top joint of the thumb > 45°	6 (6.6)	4 (12.9)	2 (12.5)	6 (12.8)
Hypermobility of proximal thumb joint	5 (5.5)	3 (9.7)	3 (18.8)	6 (12.8)
Proximal thumb hyperextensibility	32 (35.2)	8 (25.8)	7 (43.8)	15 (31.9)
Three tendons in the wrist	35 (38.5)	8 (25.8)	4 (25)	12 (25.5)
Left-handedness	8 (8.8)	1 (3.2)	0 (0)	1 (2.1)
Index finger longer than the ring finger	24 (26.4)	9 (29)	4 (25)	13 (27.7)

In Table 3 statistical interpretation of observable recessive human traits frequencies among tested subgroups was presented. There were 7 ORHT that significantly differed between subgroups NoAKI and risk, of which 4 (Blonde Hair, Double Hair Whorl, Right Thumb over Left Thumb, Top Joint of the Thumb > 45°) were significantly more frequent in the risk subgroup. There were 13 ORHT that significantly differed between subgroups NoAKI and Injury, of which 9 (Blonde Hair, Opposite Hair Whorl, Soft Hair, Continuous Frontal Hair line, Blue Eyes, Inability to Longitudinally tongue roll, Right Thumb over Left Thumb, Top Joint of the Thumb > 45°, and Hypermobility of proximal thumb joint) were significantly more frequent in the injury subgroup. There were 7 ORHT that significantly differed between subgroups risk and injury, of which 5 (Blonde Hair, Soft Hair, Continuous Frontal Hair line, Inability to Longitudinally tongue roll, Hypermobility of proximal thumb joint, Proximal thumb hyperextensibility) were significantly more frequent in the Injury subgroup. There were 2 ORHT that significantly differed between subgroups NoAKI and AKI (Injury + Risk), of which both (Straight Hair and Right Thumb over Left Thumb) were significantly more frequent in the AKI subgroup. There is significant difference in individual variations of 19 ORHTs between NoAKI and Risk subgroups [Σχ^2^ = 99.007; degree of freedom (df) = 18, *p* < 0.01]; between NoAKI and injury subgroups (Σχ^2^ = 143.615; df = 18, *p* < 0.01); between risk and injury subgroups (Σχ^2^ = 166.308; df = 18, *p* < 0.01); between AKI and NoAKI subgroups (Σχ^2^ = 4.531; df = 6, *p* = 0.605) (Tables 2, 3). According to univariate logistic regression analysis (ULRA) and multivariate logistic regression analysis (MLRA), for the subgroup of patients that had the degree of severity-Risk-according to RIFLE criteria vs. NoAKI subgroup the most significant predictors were ORHT—Inability to Longitudinally Tongue Roll (MLRA: OR-2.86) and Blond Hair (MLRA: OR-3.03) (Table 3). According to ULRA between subgroups Risk and Injury, the most significant predictor for Risk was ORHT-Inability to Longitudinally Tongue Roll (OR-12.50) (Table 2). According to ULRA and MLRA, for the subgroup of patients that had the degree of severity—AKI- according to RIFLE criteria vs. NoAKI subgroup the most significant predictors were ORHT—Right Thumb over Left Thumb (MLRA: OR-2.20) and Blond Hair (MLRA: OR-2.60) (Table 3).

**TABLE 3:** Statistical interpretation of observable recessive human traits frequencies among tested subgroups.

Observable recessive human traits	NoAKI/Risk		NoAKI/Injury		Risk/Injury		NoAKI/Risk + Injury	
	χ^2^	ULRA OR (95% CI)	χ^2^	ULRA OR (95% CI)	χ^2^	ULRA OR (95% CI)	χ^2^	ULRA OR (95% CI)
Blond hair	35.252**	0.29 (0.11–0.722)*	5.984*	0.55 (0.15–1.94)	6.179*	1.89 (0.49–7.26)	6.342*	2.839 (1.238–6.508)*
Straight hair	1.600	0.71 (0.31–1.60)	2.208	0.67 (0.23–1.94)	0.220	0.944 (0.28–3.18)	1.042	1.444 (0.712–2.931)
Double hair whorl	8.404**	0.33 (0.04–2.42)	2.200	–	3.250	–	0.466	1.978 (0.270–14.504)
Opposite hair whorl orientation	2.255	0.68 (0.25–1.85)	13.275**	0.43 (0.13–1.43)	2.883	0.64 (0.17–2.48)	1.612	1.737 (0.736–4.098)
Soft hair	1.376	1.41 (0.51–3.86)	5.883*	0.56 (0.18–1.72)	12.811**	0.40 (0.10–1.54)	0.001	1.014 (0.452–2.275)
Continuous frontal hair line	1.168	1.59 (0.32–7.80)	8.001**	0.48 (0.11–1.99)	15.661**	0.30 (0.04–2.01)	0.019	1.085 (0.342–3.442)
Attached ear lobe	5.531*	3.70 (0.45–30.18)	11.000**	–	1.600	–	3.318	0.176 (0.022–1.420)
Ear without Darwinian notch	0.137	1.13 (0.34–3.75)	9.410**	2.50 (0.30–20.57)	23.299**	2.22 (0.23–21.74)	0.364	0.714 (0.238–2.14)
Blue eyes	0.277	0.87 (0.38–1.98)	5.030*	0.56 (0.19–1.63)	2.457	0.64 (0.19–2.16)	0.647	1.337 (0.659–2.712)
Speaking deficiency-gutorral “r”	0.182	0.84 (0.16–4.58)	0.116	0.87 (0.10–8.00)	0.006	1.03 (0.09–12.35)	0.045	1.173 (0.268–5.135)
Inability to longitudinally tongue roll	13.731**	3.21 (1.30–7.92)*	15.521**	0.26 (0.07–0.97)	78.638**	0.08 (0.02–0.36)**	0.807	0.724 (0.357–1.468)
Inability to transversally tongue roll	0.555	1.20 (0.49–2.93)	2.069	1.48 (0.44–4.96)	0.596	1.23 (0.31–4.84)	0.407	0.777 (0.358–1.686)
Right thumb over left thumb	8.799**	0.43 (0.18–1.01)	9.773**	0.41 (0.13–1.27)	0.017	0.95 (0.26–3.50)	5.421*	2.381 (1.138–4.984)*
Top Joint of the Thumb > 45°	6.014*	0.48 (0.13–1.81)	5.274*	0.49 (0.09–2.70)	0.022	1.04 (0.17–6.37)	1.487	2.073 (0.63–6.824)
Hypermobility of proximal thumb joint	3.207	0.54 (0.12–2.42)	32.162**	0.25 (0.05–1.18)	6.471*	0.46 (0.08–2.62)	2.234	2.517 (0.726–8.731)
Proximal thumb hyperextensibility	2.510	1.56 (0.63–3.88)	2.101	0.70 (0.24–2.05)	9.977**	0.45 (0.13–1.60)	0.146	0.864 (0.409–1.828)
Three tendons in the wrist	4.189*	1.80 (0.72–4.46)	4.734*	1.88 (0.56–6.27)	0.025	1.04 (0.26–4.18)	2.307	0.549 (0.251–1.197)
Left-handedness	3.564	2.89 (0.35–24.10)	8.800**	–	1.600	–	2.257	0.226 (0.027–1.860)
Index finger longer than the ring finger	0.256	0.88 (0.35–2.16)	0.074	1.07 (0.32–3.65)	0.596	1.23 (0.31–4.84)	0.026	1.067 (0.484–2.355)
Σ**χ^2^**	99.007 **	–	143.615 **	–	166.308 **	–	4.531	–
		**MLRA OR (95% CI)**		**MLRA OR (95% CI)**		**MLRA OR (95% CI)**		**MLRA OR (95% CI)**
Blond hair		3.033 (1.178–7.812)*						2.597 (1.115–6.047)*
Inability to longitudinally tongue roll		0.350 (0.139–0.882)*						
Right thumb over left thumb								2.198 (1.035–4.668)*

*p < 0.05; **p < 0.01, ULRA, univariate logistic regression analysis; MLRA, multivariate logistic regression analysis.

In Table 4 it is shown that there is no significant difference in mean values of ORHTs between NoAKI and risk subgroups (*z* = −0.490, *p* = 0.624), as well as between risk/injury subgroups (*z* = –1.443, *p* = 0.150). However, there is a significant increase in the mean value of ORHTs for injury subgroup compared to the NoAKI subgroup (*z* = –2.057, *p* = 0.039). Also, there is no significant difference in the mean value of ORHTs for the AKI subgroup compared to the NoAKI subgroup (*z* = –1.271, *p* = 0.204). Variability decreased proportionally to the increase in the severity of AKI (V_*NoAKI*_ = 32.81%, V_*Risk*_ = 30.92%, V_*Injury*_ = 28.62%, V_*Risk+Injury*_ = 29.98%).

**TABLE 4:** Frequencies of observable recessive human traits in the different subgroup.

Observable recessive human traits	NoAKI	Risk	Injury	Risk + Injury
2	8 (8.8%)	3 (9.7%)	0	3 (6.4%)
3	20 (22%)	4 (12.9%)	1 (6.3%)	5 (10.6%)
4	19 (20.9%)	7 (22.6%)	5 (31.3%)	12 (25.5%)
5	21 (23.1%)	11 (35.5%)	3 (18.8%)	14 (29.8%)
6	18 (19.8%)	5 (16.1%)	3 (18.8%)	8 (17%)
7	3 (3.3%)	1 (3.2%)	2 (12.5%)	3 (6.4%)
8	2 (2.2%)	0	2 (12.5%)	2 (4.3%)
Mean ± SD	4.42 ± 1.45	4.56 ± 1.41	5.38 ± 1.54	4.77 ± 1.43
Median with IQR	4 (3–6)	5 (4–5)	5 (4–6.75)	5 (4–6)

SD, standard deviation; IQR, interquartile range; subgroup NoAKI: N = 91, MV ± SDorht/19 = 4.42 ± 1.45; subgroup risk: N = 31, MV ± SDorht/19 = 4.56 ± 1.41; subgroup injury: N = 16, MV ± SDorht/19 = 5.38 ± 1.54; subgroup AKI (injury + risk): N = 47, MV ± SDorht/19 = 4.77 ± 1.43. V_NoAKI_ = 32.81%, V_Risk_ = 30.92%, V_Injury_ = 28.62%, V_Risk+Injury_ = 29.98%; NoAKI/Risk (z = −0.490, p = 0.624); NoAKI/Injury (z = −2.057, p = 0.039); Risk/Injury (z = −1.443, p = 0.150); NoAKI/Risk + Injury (z = −1.271, p = 0.204).

## Discussion

As it is so far known, very few biochemical processes are determined by known gene locations which depict the complexity and sensitivity of genetic homozygosity estimation for certain gene loci (7, 11, 14). Having said that, it is important to stress that above-mentioned processes could be used to unveil basic changes in certain groups of individuals with heterogenous pathological or physiological conditions, which demonstrates a different degree of adaptation to changes in physiological capabilities and capacities caused by multifactorial stimuli (6–9, 11–14).

Our findings demonstrated an increased degree of genetic homozygosity along with a decrease in variability in the subgroups of the different degrees of severity expression of the AKI after cardiac surgery compared to those that did not develop AKI, also in the overall number of patients that developed any degree of AKI compared to patients that did not. It was also demonstrated that some of these genetic homozygotic traits might be considered a potential predictor for the development of AKI after cardiac surgery.

The complexity of onset and severity degree of post-cardiac surgery AKI justifies the multifactorial model of pathophysiological mechanism. In line with this, a systemic review by Vilander et al. (10) stated that different groups of genes (inflammatory, vasomotor regulation, and other genes) have different extents of association related to the onset of AKI.

Despite the large number of studies (17–22) there are still conflicting findings relating to connection between genetic predisposition and AKI. Therefore, in this research we aimed to investigate the population genetic approach of defining clearly expressed ORHTs as a measure of genetic homozygosity in cardiac surgery patients that have developed AKI after the procedure. Thus, the results obtained in this study could be referred to as hypothesis-generating rather than hypothesis-confirming regarding the possible association of the degree of genetic homozygosity and onset of AKI as well as its severity and degree of expression. Previous studies on morphogenetic levels have shown an increased degree of recessive homozygosity in patients with vascular events (cardiac and cerebrovascular) (6–8).

Increased degree of recessive homozygosity along with decreased variability in AKI patients from our study could assume the existence of a possible correlation among different polygenic combinations that might affect regulatory processes of resistance to AKI onset and the degree of AKI severity.

In addition to this, our findings stressed out different ORHTs as predictors not only for the onset of AKI but also for the expression of its severity degree. Assuming preferential genotypes might exist, our hypothesis generating model could refer, to the certain degree, to an easier expression of AKI due to altered state in a specific multifactorial setting with a presence of such predictors. Moreover, increased genetic homozygosity, which presumably corresponds to the increase in genetic loads, might be the cause of easier expression of AKI.

In the analysis of individual ORHT distribution in our study, we have demonstrated the difference in the distribution of certain phenotype traits among patients with the presence and different severity degrees of AKI after cardiac surgery. This could imply that preferential phenotypes might lead to the changes in predisposition risk not only to the development of AKI but to the degree of severity itself. Considering this, our findings might argue for the potential multifactorial etiopathogenesis due to the presumably modified individual adaptability potential to the specific extrinsic factors, which could, to a certain degree, provoke the easier expression of AKI (9).

There are several limitations to this study.

The first limitation refers to the number of tested individuals, thus findings on larger samples would increase the sensitivity of the results.

The second limitation is the fact that this study is conducted on individuals only from Serbian population, and that this is a single-center study. Taking into consideration that specific socio-economic and genetic variations may exist among different populations, further studies conducted on different populations are needed.

The third potential limitation could also be considered the use of GFR in evaluation of AKI. Renal dysfunction after cardiac surgery has a major role in both operative and long-term mortality. Even patients with preoperative normal renal function can, due to the specificity of cardiac surgery, develop an acute renal injury. One of the important things that distinguish cardiac surgery as a high risk for AKI is the use of The effect of CPB on renal function is complex and numerous—non-pulsatile and low flow, low pressure perfusion with consequential reduced renal perfusion, impaired autoregulation, systemic hypotension, hemodilution, activation of inflammatory response. Even when patients are hemodynamically stable monitoring-wise, there could be a decrease in renal blood flow. Due to capillary leak caused by CPB, patients will be overloaded with fluids in order to keep optimal cardiac output. This effect can last up to 12 h after the procedure. After that, diuretics could be needed to eliminate excess fluids which could compromise the already affected heart (23, 24).

Some authors argue that GFR provides a better estimate of renal reserve and capacity of kidneys to deal with surgical stress and the fact that serum creatinine could stay within normal range even when GFR decreases in half. All mentioned above together with the fact that routine evaluation of biomarkers for tubular damage such as neutrophile gelatinase-associated lipocalin (NGAL), Kidney Injury Molecule 1 (KIM1), interleukin-18 (IL-18) are not available in all centers including our hospital, the use of GFR to evaluate acute kidney injury in patients after cardiac surgery is more timely than serum creatinine level (24). The early detection of kidney injury is of the essence for preventing its progression and for successful management.

## Conclusion

Our findings pointed to a higher degree of recessive homozygosity and decreased variability in AKI patients vs. NoAKI individuals. This could imply that different genes with presumably modified expression might be present in different proportions, thus facilitating processes that govern easier development and severity degree expression of AKI in patients after cardiac surgery.

## Data availability statement

The original contributions presented in this study are included in the article/supplementary materials, further inquiries can be directed to the corresponding author.

## Ethics statement

The studies involving human participants were reviewed and approved by Ethics Committee of University Clinical Center of Serbia. The patients/participants provided their written informed consent to participate in this study.

## Author contributions

RK, NK-K, and BK contributed to the study design, data collection, and manuscript writing. BK, JČ, and VV contributed to data collection and statistical analysis. DT, VM, and MV contributed to data interpretation. All authors approved the final version of the manuscript.
